# Brain structure and symptom dimensions in borderline personality disorder

**DOI:** 10.1192/j.eurpsy.2019.16

**Published:** 2020-02-07

**Authors:** Igor Nenadić, Annika Voss, Bianca Besteher, Kerstin Langbein, Christian Gaser

**Affiliations:** 1 Department of Psychiatry and Psychotherapy, Philipps University Marburg & Marburg University Hospital/UKGM, Marburg, Germany; 2 Center for Mind, Brain, and Behaviour (CMBB), Marburg, Germany; 3 Department of Psychiatry and Psychotherapy, Jena University Hospital, Jena, Germany; 4 Department of Neurology, Jena University Hospital, Jena, Germany

**Keywords:** borderline personality disorder, Borderline Symptom List, orbitofrontal cortex, voxel-based morphometry, Young Schema Questionnaire

## Abstract

**Background.:**

Borderline personality disorder (BPD) presents with symptoms across different domains, whose neurobiology is poorly understood.

**Methods.:**

We applied voxel-based morphometry on high-resolution magnetic resonance imaging scans of 19 female BPD patients and 50 matched female controls.

**Results.:**

Group comparison showed bilateral orbitofrontal gray matter loss in patients, but no significant changes in the hippocampus. Voxel-wise correlation of gray matter with symptom severity scores from the Borderline Symptom List (BSL-95) showed overall negative correlation in bilateral prefrontal, right inferior temporal/fusiform and occipital cortices, and left thalamus. Significant (negative) correlations with BSL-95 subscores within the patient cohort linked autoaggression to left lateral prefrontal and insular cortices, right inferior temporal/temporal pole, and right orbital cortex; dysthymia/dysphoria to right orbitofrontal cortex; self-perception to left postcentral, bilateral inferior/middle temporal, right orbitofrontal, and occipital cortices. Schema therapy-based Young Schema Questionnaire (YSQ-S2) scores of early maladaptive schemas on emotional deprivation were linked to left medial temporal lobe gray matter reductions.

**Conclusions.:**

Our results confirm orbitofrontal structural deficits in BPD, while providing a framework and preliminary findings on identifying structural correlates of symptom dimensions in BPD, especially with dorsolateral and orbitofrontal cortices.

## Introduction

Borderline personality disorder (BPD) presents with a number often heterogeneous symptoms including impulsivity, dysphoria or affective instability, repeated self-harm, and disturbed self-perception [1]. There is considerable comorbidity with affective disorders, substance abuse, eating disorders, as well as other personality disorders such as Cluster C [2,3]. Also, there is evidence for subtle neuropsychological deficits, for example, in executive functions [4]. Current research has therefore focused on a dimensional understanding of disease pathophysiology as well as identification of suitable endophenotypes [5].

Neurobiological models of BPD have evolved based on the identification of fronto-limbic dysfunction and changes in brain structure [6–8] as well as neuroendocrine changes association with oxytocin function [9]. However, there is still considerable heterogeneity across studies.

Brain structural abnormalities have been shown in several studies of BPD, but the location and extent has been under debate. Early studies using voxel-based morphometry (VBM) have suggested reductions of gray matter in the amygdala [10,11], while subsequent studies have shown reductions in the orbitofrontal cortices [12,13], the hippocampus, and the dorsolateral prefrontal cortex [14]. A recent analysis across multiple studies identified these areas as well as multiple prefrontal cortical, cingulate cortex, and insular areas [15]; however, it included only part of the published data owing, in part, due to the different methodologies used across studies.

A second major goal in morphometric studies of BPD has been the issue of relating brain structural changes to symptom patterns or disease severity. One study, while failing to identify diagnosis-related hippocampal volume reduction, showed that volumetric variation in patients might depend on disease severity [16]. Similarly, childhood abuse has been linked to prefrontal cortical changes in BPD [17]. However, other associations have been rather inconclusive or not replicated [16,18,19]. Similarly, the overlap with typically comorbid disorders has only begun to be explored, such as comparison with major depression [20], or avoidant personality disorder [21].

Several factors might contribute to the heterogeneity of findings in BPD. Some have been limited to the study of female patients [11,13,18], given that these might present more frequently in hospital settings. Studies in male BPD are relatively scarce [22,23]. Also, subgroups within BPD, as shown in the study of patients with versus without suicide attempts have been shown to differ in areas including the insula, orbitofrontal cortices, and parahippocampal cortices [24]. More importantly, psychiatric comorbidity, such as post-traumatic stress disorder (PTSD), other personality disorders, or affective disorders might impact on the extent or variation of gray matter deficits, even though the precise nature of differential effects or effect sizes is poorly understood [16,17,20,25]. Finally, one study also showed variation of group-level differences in different age groups of BPD patients [26].

In the present study, we aimed to add to the current research by providing a VBM analysis of female BPD patients compared to healthy controls as well as a correlation with symptoms. For the latter, we chose a self-rating scale, which allowed us to use a DSM-related framework for a dimensional approach. In addition, we assessed early maladaptive schemas (EMS) according to Young’s conceptualization in the Young Schema Questionnaire (YSQ). The early maladaptive schema assessment is commonly used in schema therapy approaches to treating BPD, and is based on the assumption that patients show a number of typical maladaptive “blue prints” for emotional and cognitive reactions to situations that have developed during childhood and adolescence, often as a response to (parental) neglect or maltreatment. They hence reflect persistent dysfunctional emotional reaction patterns and cognitions based on early experiences, and have been used for both diagnostic aspects in therapy planning as well as outcome of schema therapy interventions [27]. Therefore, we tested both the hypotheses of BPD-related gray matter loss in the orbitofrontal cortex (OFC), amygdala, and hippocampus, as well as relating changes in these as well as medial and lateral prefrontal areas to (a) severity of BPD symptoms and (b) severity of EMS (derived from a clinical schema therapy-based scale).

## Methods

### Study sample

We studied 19 female patients with BPD and 50 female healthy controls, all of which provided written informed consent to a study protocol approved by the ethics committee of the Medical School of the University of Jena. Groups were matched for age (mean age patients: 26.7 years, SD 6.4; mean age controls: 26.8 years, SD 5.9; analysis of variance (ANOVA) *F*_(69,1)_ = 0.001; *p* = 0.980), gender (all being female), as well as estimated premorbid intelligence quotient IQ (as estimated with the Mehrfach Wortschatzstest-B; mean score patients: 104, SD 11.2; mean score controls: 104.2, SD 12; ANOVA *F*_(69,1)_ = 0.005; *p* = 0.945) and handedness (estimated by the Edinburgh Handedness Scale [28]; ANOVA *F*_(69,1)_ = 0.711, *p* = 0.402). Diagnoses in the patient group were established by a board-certified psychiatrist (I.N.) according to DSM-IV criteria (Diagnostic and Statistical Manual of Mental Disorders) using the SCID-II screening inventory and additional interview. The patients also met DSM-5 criteria (following the conventional typology), as evaluated subsequent to the introduction of DSM-5 (German translation). At the time of study inclusion, comorbidity history in the patient cohort included: major depressive disorder, currently in remission (*n* = 9), post-traumatic stress disorder (*n* = 2), avoidant personality disorder (*n* = 2), alcohol abuse (*n* = 1), and eating disorder (*n* = 4). None of the patient had a current major depressive episode or alcohol or substance dependence. Within the patient cohort, *n* = 8 received antidepressant medication (three were on sertraline, another three on citalopram, one each on mirtazapine and doxepine, respectively). Healthy controls were recruited from the local community and had no history of psychiatric disorders or treatment. General exclusion criteria were concurrent neurological or general medical conditions, traumatic brain injury, or learning disability.

Symptoms and psychopathology in the patient group was also assessed using the Borderline Symptom List (BSL-95), German version [29]. This self-rating inventory, which considers DSM-IV criteria for BPD as well as criteria from the diagnostic interview for BPD (revised version) has been validated in several samples and versions across different languages, showing high internal reliability, short-term test–retest-reliability, as well as convergence with the above standard diagnostic criteria [30,31]. Factor analysis suggests seven subscales, which have been named self-image, affect regulation, autoaggression, dysthymia, social isolation, intrusions, and hostility [29], or alternatively self-perception, affect regulation, self-destruction, dysphoria, loneliness, intrusions, and hostility [30]. BSL-95 total and subscale scores for *n* = 18 patients (missing data for one patient) are shown in Table 1. We used the Young Schema Questionnaire (YSQ-S2) for assessment of EMS (German version, as in [32,33]), based on Jeffrey Young’s maladaptive schema model, which forms the core for schema therapy-based clinical interventions. The used version has 95 items to cover 19 maladaptive schemas.

**Table 1.** Borderline Symptom List (BSL-95) scores and Young Schema Questionnaire (YSQ-S2) scores of the BPD patient sample (*n* = 18).

	Score	SD
BSL-95 scores		
Self-perception	32.00	12.9
Affect regulation	31.56	7.5
Self-destruction	30.67	10.8
Dysphoria	31.22	6.4
Loneliness	25.00	7.9
Hostility	10.33	4.5
Intrusions	11.61	5.5
BSL total score	203.61	41.7
YSQ-S2 scores		
Emotional deprivation	21.63	5.98
Mistrust/abuse	21.74	6.16
Abandonment	22.16	4.41
Insufficient self-control	26.53	4.30

### Magnetic resonance imaging acquisition and analysis

High-resolution T1-weighted magnetic resonance imaging (MRI) scans were acquired on a 3 T Siemens Tim Trio scanner (Siemens, Erlangen, Germany) with a MPRAGE sequence (1 mm × 1 mm × 1 mm voxel resolution; TR 2,300 ms, TE 3.03 ms; flip angle 9°; sagittal acquisition of 192 contiguous slices; field-of-view 256 mm). All images passed visual inspection for artifacts, as well as the automated quality assurance protocol implemented in VBM8.

For postprocessing of MRIs, we used the VBM8 toolbox (http://dbm.neuro.uni-jena.de/vbm), implemented in SPM8 (Statistical Parametric Mapping software, Institute of Neurology, London, UK; http://fil.ion.ucl.ac.uk/spm). VBM8 makes use of the diffeomorphic image registration algorithm (DARTEL) of SPM8 [34] in the general framework of the optimized VBM approach [35]. In the process of segmentation, an internal threshold for gray matter of 0.2 was applied, which is more conservative than commonly applied thresholds (e.g., 0.1), restricting artifacts at the gray matter boundaries. A 12 mm FWHM (full-width at half-maximum) Gaussian filter was used for smoothing.

Statistical analysis of VBM results was done in SPMs general linear model (GLM) framework model with three sets of analyses. First, a (categorical) group comparison of the BPD patient cohort versus healthy control cohort to identify BPD-related structural changes was computed, hypothesizing changes in the hippocampus/medial temporal lobe, as well as orbitofrontal and cingulate cortices, as shown in the previous imaging studies. Second, the total BSL score as an indicator of overall BPD-related psychopathology was correlated (within the BPD patient sample) to gray matter across the entire brain, again hypothesizing severity-related correlations in the above areas. Third, an exploratory analysis with correlations of each of the BSL subscales, that is, self-perception, affect regulation, autoaggression/self-destruction, dysthymia/dysphoria, social isolation/loneliness, intrusions, and hostility was performed.

An additional exploratory analysis was based on patient self-ratings using the YSQ (German adaptation of YSQ-2). From this, we selected the schemas of emotional deprivation, mistrust/abuse, abandonment, and insufficient self-control.

For each GLM, age was used as a covariate in order to eliminate any potential effect of this variable (gender was omitted as all participants were female), and height threshold was set to *p* < 0.001 (uncorrected) with an additional extent threshold *k* (depending on the SPM resolution element (resel) estimate in respective analyses).

## Results

### Group comparison of BPD versus healthy controls

**Figure 1.**
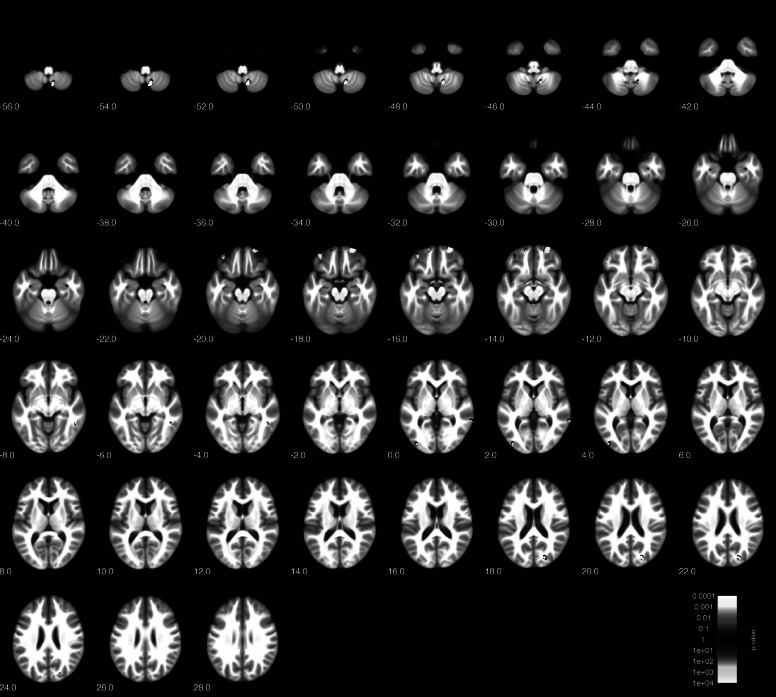
Voxel-based morphometry (VBM) group comparison of borderline personality disorder (BPD) patients and healthy controls (HC) at *p* < 0.001 (uncorrected).

Comparison of BPD patients with healthy controls showed gray matter reduction (*p* < 0.001, uncorrected; expected voxels per cluster *k* = 226 voxels) in the right orbitofrontal cortex in patients (maximum intensity voxel at *x*/*y*/*z* coordinates 26; 60; −18; cluster size *k* = 299 voxels), but not in hippocampus or cingulate areas (see Table 2 and Figure 1 for an overview of clusters at *p* < 0.001 with *k* = 50 thresholds for full overview). Additionally, we found second orbitofrontal cluster in the left hemisphere, more laterally (maximum voxel: 36; 47; −20; *k* = 95 voxels), which was significant at *p* < 0.001, but failed to reach the extent threshold.

**Table 2.** Overview of voxel-based morphometry (VBM) group comparison of borderline personality disorder (BPD) patients and healthy controls (HC) at *p* < 0.001 (uncorrected), showing clusters with *k* > 50 voxels.

	*k*	*T* score	MNI co-ordinates of maximum voxel
Gray matter HC > BPD			
Right middle/superior frontal gyrus, pars orbitalis	299	4.30	26; 60; −18
Left inferior/middle frontal gyrus, pars orbitalis/orbitofrontal cortex	95	3.52	−36; 47; −20
Gray matter HC < BPD			
Right superior occipital gyrus	57	3.51	21; −81; 19
Right cerebellum (VIII, IX)	218	3.50	10; −64; −53

*k* represents the number of voxels in cluster.

### Correlation of gray matter with overall disease severity (BSL total score)

**Figure 2.**
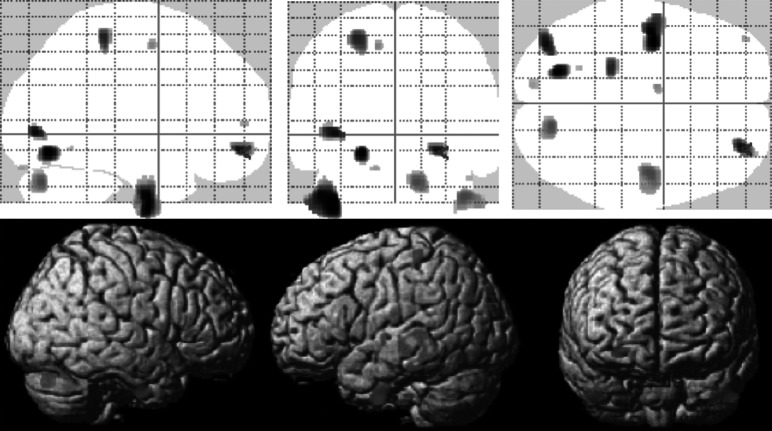
Voxel-based morphometry (VBM) correlation analysis of gray matter and BSL total score in *n* = 18 patients with borderline personality disorders (BPD) at *p* < 0.001 (uncorrected).

Correlation of gray matter with BSL total scores (within the BPD cohort) revealed significant negative correlations (*p* < 0.001, uncorrected; *k* = 148) in several prefrontal and temporal areas including left inferior temporal, lingual and middle occipital cortices, left postcentral cortex, right inferior temporal cortex, right cerebellar hemisphere, as well as the right orbitofrontal cortex (overlapping with the cluster identified in the group comparison). Results are shown in Figure 2 and Table 3. There were no positive correlations at *p* < 0.001.

**Table 3.** Overview of voxel-based morphometry (VBM) correlation analysis of gray matter and BSL total score in *n* = 18 patients with borderline personality disorders (BPD) at *p* < 0.001 (uncorrected), showing clusters with *k* > 50 voxels.

	*k*	T-Wert	MNI co-ordinates of maximum voxel
Left inferior temporal gyrus	816	5.46	−39; −6; −42
Left lingual gyrus	178	5.28	−21; −70; −11
Left medial occipital gyrus	154	4.84	−38; −78; 1
Right middle/superior frontal gyrus, pars orbitalis	165	4.82	27; 51; −9
Left postcentral gyrus	204	4.53	−22; −34; 60
Right inferior temporal gyrus	365	4.23	45; −9; −38
Right cerebellum (I, II)	239	4.22	15; −75; −29

*k* represents the number of voxels in cluster.

### Correlation of gray matter with symptom dimensions

Exploratory single symptom item correlations revealed several significant correlations, mostly negative correlations, including several prefrontal clusters.

**Figure 3.**
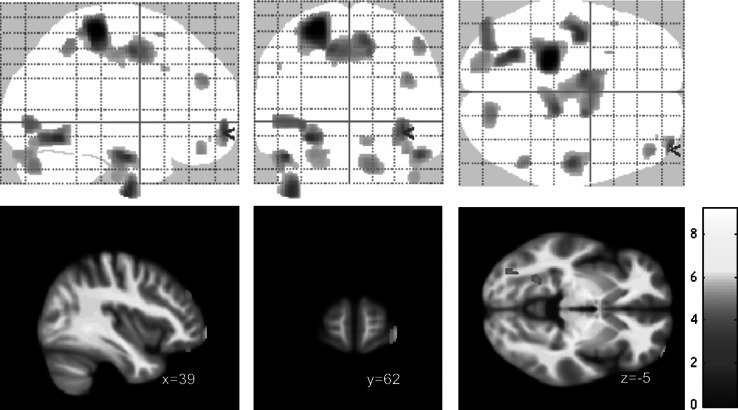
Voxel-based morphometry (VBM) correlation analysis of gray matter and BSL self-perception (or self-image) subscore in *n* = 18 patients with borderline personality disorders (BPD) at *p* < 0.001 (uncorrected). Note that the precentral/postcentral cortex cluster (k=1928) also survived corrected for multiple comparisons at p<0.05 FWE.

Of note, we found a strong signal for a negative correlation between the subscore self-perception and a cluster in the left precentral and postcentral cortex (maximum voxel: −24; −31; 63; *k* = 1,928), part of which also survived correction for multiple comparisons at *p* < 0.05 with FWE correction (Figure 3).

**Figure 4.**
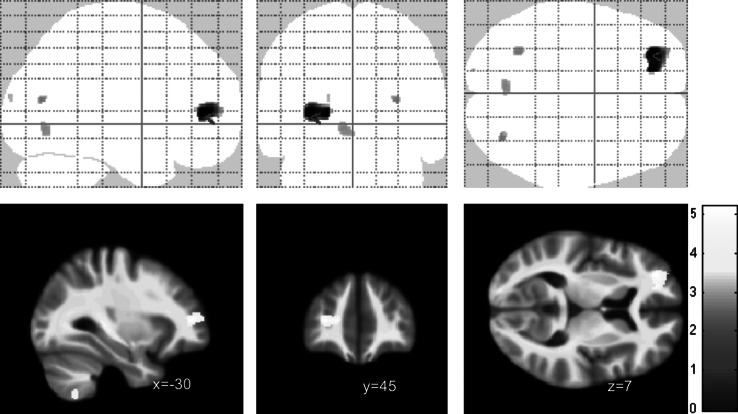
Voxel-based morphometry (VBM) correlation analysis of gray matter and BSL dysphoria subscore in *n* = 18 patients with borderline personality disorders (BPD) at *p* < 0.001 (uncorrected).

Across the BSL-subscores, we found negative correlations with orbitofrontal volumes only for the self-perception subscore in two right OFC clusters (maximum voxels 39; 62; −5, *k* = 183 and 40; 45; −21, *k* = 70 voxels, respectively) and for the autoaggression subscore (maximum voxel 26; 52; −2, *k* = 136), but not the other subscores. BSL dysthymia sub scale showed a correlation in the left middle frontal gyrus (Figure 4). Some subscores like intrusions and hostility did not show any correlations at all (neither negative nor positive).

### Correlation of gray matter with maladaptive schemas (YSQ)

Correlation analyses with YSQ-S2-assessed maladaptive schemas in patients (*p* < 0.001, uncorrected) showed a negative correlation between gray matter and emotional deprivation schema for two bilateral parahippocampal clusters (*p* < 0.001, *k* > 163 voxels; maximum voxels at −28; −9; 30, *k* = 709 voxels, and 26; −21; −32, *k* = 288 voxels, respectively). In addition, there was a positive correlation between insufficient self-control and right inferior fusiform/occipital cortex (45; −68; −18; *k* = 317 voxels). Although other correlations failed to reach the *k* extent threshold level, we noted a positive correlation for the mistrust schema with bilateral calcarine/lingual voxel clusters.

### Post hoc power analysis

Given the limited size of the patient sample, we performed a post hoc power analysis using G*Power 3.1. Based on samples of different sizes (allocation ratio 2.63 for 19 patients vs. 50 controls), a two-sample *t*-test at alpha error probability of 0.05 and power 0.8 would detect larger effects sizes of *d* = 0.7 in a sample of 18 versus 48 subjects.

## Discussion

Our study aimed to identify group difference in regional brain structure (using VBM) between female patients with BPD and healthy controls. Although we found evidence for volume reduction in the orbitofrontal cortex, our study failed to find structural changes in medial temporal lobe structures and particularly the hippocampus and amygdala. On the other hand, our symptom correlations provide evidence for a link between orbitofrontal deficits and alterations of self-perception and autoaggression (Figure 3).

Although there are now a few structural brain imaging studies in BPD, there is still little consensus on the basic pattern characterizing this disorder [8,36]. Our orbitofrontal cortical finding adds to evidence implicating this area in BPD [6,13]. So far, however, it is unclear whether this change relates to particular symptom correlates or facets of the disease phenotype. Patients with BPD show impairment in neurocognitive tasks requiring balanced decision-making, which relies (in part) on orbitofrontal cortical integrity [37]. There is also increasing evidence from functional MRI studies, especially those using resting state fMRI, that BPD is associated with aberrant activity in the OFC, both in single studies using different methodological approaches to data analysis [38–40] as well as a recent meta-analysis of resting state fMRI studies [15,41]. On a symptom level, functional studies have suggested that orbitofrontal recruitment varies according to suicide attempt history in BPD [42].

Our own findings appear to suggest that self-perception and auto-aggression would be linked to OFC volumes. Overall, this is also in line with a fronto-limbic system theory of BPD [10], which could integrate both aspects of rapidly changing emotional states as well as persistent alterations of self-referential functions [8,43]. However, our finding needs to be taken with caution, given that restriction of findings using extent thresholding is less conservative than peak-level-based correction.

Interestingly, we did not find gray matter reductions in the medial temporal lobe, especially amygdala and hippocampus. Recent studies have discussed that this feature seen in some of the earlier studies of MR morphometry in BPD might be related to particular subgroups or comorbidities such as post-traumatic stress disorder [16,44,45]. This also points to comorbidities as a main source of variance across different BPD studies. Although our study sample showed a varied combination of comorbid disorders, it is fair to say that this pattern is fairly typical of those seen in BPD across the disorder [1]. It thus still remains a major task to untangle these comorbidities. Yet, inclusion of patients who only suffer from BPD without comorbid conditions would seem to introduce a grave selection bias, rendering such a sample unlikely to be representative of BPD in general.

Finally, our exploratory analyses provide clues on which different facets of BPD might link to different brain structures. We used two different self-rating instruments, one based on the DSM symptoms (BSL) and one based on the clinically relevant schema therapy-based assessment of EMS (YSQ). Although these results are preliminary, they suggest that different aspects of BPD pathology link to orbitofrontal versus pre/postcentral cortical variations. At least one YSQ finding also suggests that cognitive/emotional schemas developing early (in this case emotional deprivation) render medial temporal lobe structures (i.e., parahippocampal cortices) prone to changes manifesting in persistent changes of emotional states.

Our study is mainly limited by its sample size, which although similar to several recent MR morphometry analyses, is prone to false positives, in particular for correlation analyses in the exploratory part. Also, although antidepressants do not seem to induce changes similar to those seem with antipsychotics, we cannot exclude an effect of medication to have interfered with volumetric measurements. Although further replication is warranted, our findings do stress the necessity to differentiate brain structural patterns not only according to symptom structure, but possibly also to persistent features inherent in many BPD patients, which might be captured using alternative tools like the YSQ. This might then also provide a link to understanding changes of brain volumes over time, be it over the course of long-term psychotherapy or spontaneous changes in the course of BPD.
